# Performance of scoring systems for predicting mortality after cardiac surgery in the elderly

**DOI:** 10.47487/apcyccv.v6i1.459

**Published:** 2025-02-12

**Authors:** Arturo M. Ruiz-Beltrán, Gerardo Chacón-Loyola, Ricardo L. Barajas-Campos, Alejandro Alcaraz-Guzmán, Manuel A. Montoya-Hernández, Leonardo D. Alcázar-Flores, Laura L. Rodríguez-Chávez

**Affiliations:** 1 Division of Cardiology, Instituto Nacional de Cardiología Ignacio Chávez, Mexico city, Mexico. Division of Cardiology Instituto Nacional de Cardiología Ignacio Chávez Mexico city Mexico

**Keywords:** Cardiac Surgical Procedures, Risk, Aged, Frail Elderly, Procedimientos Quirúrgicos Cardíacos, Riesgo, Anciano, Anciano Frágil

## Abstract

**Objective.:**

To evaluate the predictive ability of traditional risk scores and frailty assessment for 30-day mortality in elderly patients undergoing cardiac surgery.

**Materials and Methods.:**

A single-center retrospective cohort study was conducted, including elderly patients (≥75 years old) who underwent cardiac surgery between January 2005 and December 2015. EuroSCORE II, STS (Society of Thoracic Surgeons) score, Charlson Comorbidity Index, and Electronic Frailty Index scores were calculated to assess their predictive ability for 30-day mortality using receiver operating characteristic (ROC) curves and corresponding areas under the curve (AUC).

**Results.:**

A total of 203 patients were included during the study period, of whom 33% underwent isolated coronary bypass surgery. The overall 30-day mortality rate was 17.6%. Patients who died within the first 30 days exhibited higher scores on EuroSCORE II, STS, and the Electronic Frailty Index. The areas under the ROC curves were: EuroSCORE II, 0.74 (95% CI: 0.66-0.81); STS, 0.66 (95% CI: 0.57-0.77); Charlson Comorbidity Index, 0.60 (95% CI: 0.49-0.70); and Electronic Frailty Index, 0.63 (95% CI: 0.52-0.73).

**Conclusions.:**

In this cohort of elderly patients undergoing cardiac surgery, EuroSCORE II demonstrated the best performance in predicting 30-day mortality.

## Resumen

**Objetivo.:**

Evaluar la capacidad predictiva de las puntuaciones tradicionales de riesgo y de la evaluación de la fragilidad para la mortalidad a 30 días en pacientes de edad avanzada sometidos a cirugía cardíaca.

**Materiales y métodos.:**

Se realizó un estudio de cohorte histórica de un solo centro que incluyó pacientes de edad avanzada (≥75 años) sometidos a cirugía cardíaca entre enero de 2005 y diciembre de 2015. Se calcularon las puntuaciones EuroSCORE II, STS (Society of Thoracic Surgeons), índice de comorbilidad de Charlson e índice electrónico de fragilidad para evaluar su capacidad predictiva de la mortalidad a 30 días mediante curvas características operativas del receptor (ROC) y áreas bajo la curva (AUC) correspondientes.

**Resultados.:**

Durante el periodo de estudio se incluyó a 203 pacientes, de los cuales el 33% fueron sometidos a cirugía aislada de baipás coronario. La tasa global de mortalidad a los 30 días fue del 17,6%. Los pacientes que fallecieron dentro de los primeros 30 días presentaron puntuaciones más altas en EuroSCORE II, STS e índice electrónico de fragilidad. Las áreas bajo la curva ROC obtenidas fueron: EuroSCORE II, 0,74 (IC 95%: 0,66-0,81); STS, 0,66 (IC 95%: 0,57-0,77); índice de comorbilidad de Charlson, 0,60 (IC 95%: 0,49-0,70) e índice electrónico de fragilidad, 0,63 (IC 95%: 0,52-0,73).

**Conclusiones.:**

En esta cohorte de pacientes de edad avanzada sometidos a cirugía cardíaca, EuroSCORE II mostró el mejor desempeño para predecir la mortalidad a los 30 días.

## Introduction

Patients undergoing cardiac surgery are increasingly older and have a higher prevalence of comorbidities, making accurate risk assessment crucial for guiding treatment decisions and avoiding futile interventions. ^1^ Although risk scores such as EuroSCORE II and the STS score are used to predict prognosis and resource utilization, their validity for predicting mortality in elderly patients is limited, as they do not fully capture the biological status of these individuals.^2^^,^^3^ Previous studies have highlighted discrepancies between predicted and observed mortality using these scores, particularly in older adults, with significantly lower areas under the receiver operating characteristic (ROC) curve (AUROC) in the elderly compared to younger patients. ^4^^-^^6^ At our center, previous research by Rodriguez-Chavez et al. reported only borderline discriminatory power for EuroSCORE II (AUROC = 0.71). ^7^

Frailty, which is highly prevalent and strongly associated with operative mortality and prolonged hospital stays among older adults, may enhance risk stratification beyond existing scores. ^8^^,^^9^ Current guidelines from the European Society of Cardiology recommend objective frailty assessment using tools such as the Katz Index or the Essential Frailty Toolset. ^10^ The latter demonstrated superior performance in predicting 12-month mortality following aortic valve intervention (AUROC = 0.78) in the FRAILTY-AVR study. ^11^ However, significant heterogeneity exists in the reported prevalence and effect sizes across studies. ^12^ To evaluate medium- to long-term mortality, the ASCERT score and the Charlson Comorbidity Index (CCI) have been utilized; notably, the CCI has shown a significant association with mortality and rehospitalization following coronary revascularization. ^13^^,^^14^

This study aimed to assess the predictive ability of traditional risk scores and frailty assessment in predicting 30-day mortality after cardiac surgery in elderly patients.

## Materials and methods

### Study design and population

This single-center (Instituto Nacional de Cardiología Ignacio Chávez), retrospective cohort study included patients aged ≥75 years who underwent valve replacement and/or coronary artery bypass grafting (CABG) with extracorporeal circulation between January 2005 and December 2015. Patients with incomplete electronic health record data were excluded.

### Data collection and variables

Data were extracted from electronic health records and included preoperative patient characteristics, surgical details, and postoperative outcomes.

The EuroSCORE II was calculated as originally described, ^15^ incorporating age, sex, insulin-dependent diabetes mellitus, chronic lung disease, neurological or musculoskeletal dysfunction severely affecting mobility, creatinine clearance, preoperative critical status, New York Heart Association (NYHA) functional class, angina symptoms, peripheral artery disease, previous cardiac surgery, active endocarditis, left ventricular ejection fraction, recent myocardial infarction, pulmonary artery systolic pressure, urgency of the procedure, complexity of the procedure, and thoracic aorta surgery.

The STS score was computed using the online calculator version 2.0.5 (available at riskcalc.sts.org), based on the 2018 update. ^16^ The score included planned surgery, demographic data, laboratory values, preoperative medications, risk factors/comorbidities, cardiac status, coronary artery disease, valve disease, arrhythmias, and previous cardiac interventions.

The Charlson Comorbidity Index (CCI) was calculated according to the original description, ^17^ including age, myocardial infarction, congestive heart failure, peripheral vascular disease, cerebrovascular disease, dementia, chronic pulmonary disease, connective tissue disease, peptic ulcer disease, liver disease, diabetes, hemiplegia, moderate to severe chronic kidney disease (CKD), solid tumor, leukemia, lymphoma, and acquired immunodeficiency syndrome.

Frailty was evaluated using the Electronic Frailty Index (EFI), which identifies older individuals with mild, moderate, or severe frailty using routinely collected data. The EFI has demonstrated predictive validity for mortality, hospitalization, and nursing home admissions. ^18^

### Study outcome

The primary outcome was 30-day mortality, defined as death occurring within 30 days postoperatively or during hospitalization.

### Statistical analysis

Data analysis was performed using IBM SPSS Statistics, Version 29.0. Figures were created using R software, Version 4.3.0. Quantitative variables were summarized as mean ± standard deviation (parametric data) or median and interquartile range (non-parametric data), as appropriate. Bivariate analysis of quantitative variables was performed using Student’s t-test (parametric data) or the Mann-Whitney U test (non-parametric data). Qualitative variables were presented as absolute frequencies and percentages, and bivariate analyses were conducted using the χ² test or Fisher›s exact test, as appropriate. The discriminatory performance of each scoring system was evaluated using receiver operating characteristic (ROC) curves and by calculating the area under the ROC curve (AUROC) with 95% confidence intervals (CI). Statistical significance was set at p<0.05, using two-tailed tests.

### Ethical aspects

The institutional ethics committee approved the study. Patient consent was waived due to the retrospective nature of the study and data anonymization.

## Results

Of the 237 patients initially identified, 24 were excluded due to incomplete data, leaving 203 patients available for analysis. The cohort had a mean age of 77.8 ± 2.7 years, and 39.2% were women. A minority of patients (19.2%) were aged ≥80 years. Over half of the patients (55.8%) were overweight or obese, and 20.1% had chronic kidney disease (CKD). Baseline characteristics are presented in Table 1, and preoperative echocardiographic data are summarized in Table 2. The mean EuroSCORE II was 4.3 ± 4.5%, STS score 3.4 ± 3.8%, CCI 4.5 ± 1.0 points, and EFI 0.18 ± 0.06.

**Table 1:** Baseline characteristics

Characteristics	Total (N = 203)	Alive at 30 days (n = 167)	Deceased at 30 days (n = 36)	***p*-value**
Age, years ^a^	77.8 ± 2.7	77.7 ± 2.6	78.1 ± 3.0	0.424^c^
Female, n (%)	80 (39.4)	57 (34.1)	23 (63.9)	<0.001^d^
BMI, kg/m^2 a^	25.5 ± 3.9	25.5 ± 3.5	25.2 ± 5.7	0.609^c^
Current/past smoking, n (%)	81 (39)	70 (41.9)	11 (30.6)	0.207^d^
Diabetes mellitus, n (%)	53 (26.1)	45 (26.9)	8 (22.2)	0.558^d^
Hypertension, n (%)	120 (59.1)	97 (58.1)	23 (63.9)	0.520^d^
GFR, mL/min/1.73 m^2 a^	71.2 ± 17.8	72.4 ± 17.5	65.5 ± 17.9	0.032^c^
Atrial fibrillation, n (%)	24 (11)	16 (9.6)	8 (22.2)	0.033^e^
COPD, n (%)	16 (7.8)	11 (6.6)	5 (13.9)	0.140^e^
Previous stroke, n (%)	12 (5.8)	10 (6)	2 (5.6)	0.921^e^
Peripheral artery disease, n (%)	4 (1.9)	2 (1.2)	2 (5.6)	0.088^e^
ACS, n (%)	29 (14.2)	22 (13.2)	7 (19.4)	0.329^d^
Type of ACS, n (%)				
STEMI	7 (3.4)	6 (3.6)	1 (2.8)	0.457^e^
NSTEMI	22 (10.8)	16 (9.6)	6 (16.7)	
Chronic coronary syndrome, n (%)	53 (26.1)	45 (26.9)	8 (22.2)	0.558^d^
Left main or three-vessel disease, n (%)	65 (32)	55 (32.9)	10 (27.8)	0.548^d^
Endocarditis, n (%)	5 (2.4)	5 (3)	0 (0)	0.293^e^
STS score ^b^	2.3 (1.5-3.9)	2.1 (1.4-3.7)	3.5 (2.2-5.2)	<0.001^f^
EuroSCORE II ^b^	2.7 (1.7-4.4)	2.5 (1.7-3.8)	5.0 (2.8-8.1)	<0.001^f^
Charlson Comorbidity Index ^b^	4 (4-5)	4 (4-5)	4.5 (4-5.5)	0.114^f^
Electronic Frailty Index ^b^	0.19 (0.13-0.22)	0.19 (0.13-0.22)	0.21 (0.18-0.25)	0.007^f^

a Data expressed as mean ± standard deviation.

b Data expressed as median (IQR).

c Student's t-test.

d χ² test.

e Fisher's exact test.

f Mann-Whitney U test.

ACS, acute coronary syndrome; BMI, body mass index; COPD, chronic obstructive pulmonary disease; GFR, glomerular filtration rate; IQR, interquartile range; NSTEMI non-ST-elevation myocardial infarction; STEMI, ST-elevation myocardial infarction; STS, Society of Thoracic Surgeons.

**Table 2:** Echocardiographic characteristics before surgery

Characteristics	Total (N = 203)	Alive at 30 days (n = 167)	Deceased at 30 days (n = 36)	***p*-value** ^b^
LVEF, % ^a^	55.2 ± 10.7	57.8 ± 11.1	57.8 ± 9.2	0.970
TAPSE, mm ^a^	18.1 ± 4.4	19.4 ± 4.3	18.3 ± 4.7	0.281
Tricuspid S’ peak systolic velocity, cm/sec ^a^	10.9 ± 4.58	12.8 ± 3.4	13.9 ± 7.4	0.310
RV fractional area change, % ^a^	38.6 ± 7.7	40.6 ± 7.6	39.4 ± 8.2	0.510
PASP, mmHg ^a^	32.1 ± 16.1	36.6 ± 15.8	40.7 ± 17.1	0.172

a Data expressed as mean ± standard deviation.

b Student's t-test.

LVEF, left ventricular ejection fraction; RV, right ventricle; TAPSE, tricuspid annular plane systolic excursion; PASP pulmonary artery systolic pressure.

Surgical procedures performed (Table 3) included isolated CABG (33%), valve replacement (58.8%), and combined CABG and valve replacement (7.8%). A higher proportion of combined procedures was observed among patients who died within 30 days. Multiple valve replacements were conducted in 6.6% of the patients. No patient required emergency surgery.

**Table 3:** Procedural characteristics

Characteristics	Total (N = 203)	Alive at 30 days (n = 167)	Deceased at 30 days (n = 36)	***p*-value**
Type of surgery, n (%)				0.002^b^
AVR	92 (45.3)	76 (45.5)	16 (44.4)	
MVR	18 (8.8)	15 (9)	3 (8.3)	
AVR and MVR	4 (1.9)	3 (1.8)	1 (2.8)	
MVR and TVR	5 (2.4)	3 (1.8)	2 (5.6)	
AVR and TVR	2 (0.9)	0 (0)	2 (5.6)	
CABG	67 (33)	60 (35.9)	7 (19.4)	
CABG and AVR	13 (6.4)	9 (5.4)	4 (11.1)	
CABG and MVR	2 (0.9)	1 (0.6)	1 (2.8)	
Timing of surgery, n (%)				
Elective	114 (56.1)	102 (61.1)	12 (33.3)	0.002^b^
Urgent	89 (43.8)	65 (38.9)	24 (66.7)	
Type of valve, n (%)				
Bioprosthetic valve	130 (95)	102 (95.3)	28 (96.5)	0.776^b^
Mechanical valve	6 (4.4)	5 (4.6)	1 (3.4)	
Size of the valve, mm ^a^	22.5 ± 2.8	22.5 ± 2.8	22.7 ± 3.1	0.735^c^
Surgical time, minutes ^a^	222 ± 102	208.9 ± 91	287.11 ± 127.4	<0.001^c^
Aortic cross-clamp time, minutes ^a^	69 ± 36	66.4 ± 33	98.0 ± 39	<0.001^c^

a Data expressed as mean ± standard deviation.

b Fisher's exact test.

c Student's t-test.

AVR, aortic valve replacement; CABG, coronary artery bypass grafting; MVR, mitral valve replacement; TVR, tricuspid valve replacement.

The overall 30-day mortality rate was 17.6%. Patients who died within 30 days had significantly higher STS scores [3.5 (2.2-5.2) vs. 2.1 (1.4-3.7), p < 0.001], EuroSCORE II scores [5.0 (2.8-8.1) vs. 2.5 (1.7-3.8), p < 0.001], and EFI scores [0.21 (0.18-0.25) vs. 0.19 (0.13-0.22)] compared to survivors.

**Figure 1:**
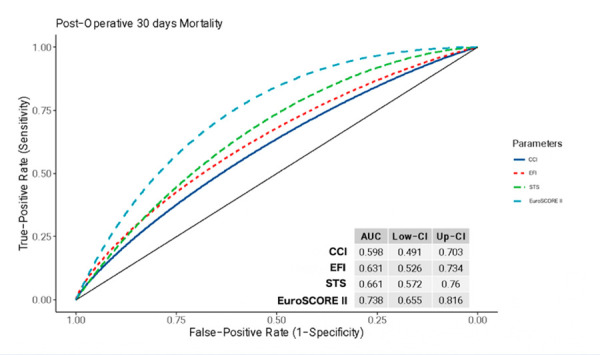
AUROC to evaluate the different scores for postoperative 30-day mortality.

AUC, area under the curve; CCI, Charlson Comorbidity Index; CI, confidence interval; EFI, Electronic Frailty Index; STS, Society of Thoracic Surgeons.

The AUROCs for predicting 30-day mortality (Figure 1) were as follows: EuroSCORE II (0.74, 95% CI 0.66-0.82), STS score (0.67, 95% CI 0.57-0.76), CCI (0.60, 95% CI 0.49-0.70), and EFI (0.63, 95% CI 0.53-0.73). Results from a combined model are provided in Table 4.

**Table 4:** AUROC of combined preoperative scores for predicting 30-day mortality

Model	AUROC	95% CI
CCI + EuroSCORE II	0.70	0.61-0.71
CCI + STS score	0.62	0.52-0.62
CCI + EuroSCORE II + STS score	0.71	0.61-0.70
EFI + EuroSCORE II	0.70	0.60-0.70
EFI + STS score	0.65	0.55-0.65
EFI + EuroSCORE II + STS score	0.70	0.60-0.70
CCI + EFI + EuroSCORE II	0.70	0.61-0.70
CCI + EFI + STS score	0.64	0.55-0.64
CCI + EFI + EuroSCORE II + STS score	0.71	0.61-0.71

AUROC, area under ROC curve; CCI, Charlson Comorbidity Index; CI, confidence interval; EFI, Electronic Frailty Index; STS, Society of Thoracic Surgeons.

## Discussion

This study investigated the predictive accuracy of several risk scores for 30-day mortality in older adults undergoing cardiac surgery. The main findings were that the overall 30-day mortality rate in our cohort was relatively high, the EuroSCORE II exhibited the best discriminatory performance for predicting 30-day mortality among older adults, and incorporating frailty into a combined predictive model did not enhance the discriminatory ability of the evaluated tools.

As the global population ages, a sustained increase in cardiac surgeries among older patients is anticipated. In a 15-year national series, nearly half of the patients were aged ≥70 years, and there was a three-fold increase in the proportion of octogenarian patients. ^19^ Although age is one of the most robust risk factors for adverse outcomes after cardiac surgery, the high 30-day mortality rate (17.6%) observed in our cohort warrants further investigation. ^20^ This elevated mortality could be attributed to several factors. First, our study population included a high proportion of patients undergoing urgent and combined procedures. Second, our cohort had a relatively high percentage of women, a population previously shown to experience higher rates of adverse events, including short-term mortality after cardiac surgery. ^21^ Third, our findings align with reported mortality rates in comparable Hispanic cohorts but contrast with reports from other centers showing lower mortality rates. ^1^^,^^22^^-^^24^

EuroSCORE II consistently demonstrated superior performance for predicting 30-day mortality, achieving an AUROC of 0.74. This finding aligns with previous studies reporting comparable AUROC values in elderly cardiac surgery patients; however, it contrasts with previous reports indicating AUROCs below 0.7 in patients aged ≥70 years undergoing various cardiac surgeries.^6^^,^^7^^,^^25^ Nonetheless, EuroSCORE II has been noted to overestimate mortality risk in octogenarians at high surgical risk. ^26^ Given that most of our cohort was younger than 80 years, this limitation may not entirely explain our observations.

Although multicomponent frailty instruments have been shown to outperform EuroSCORE II in predicting functional survival, our study found that EuroSCORE II outperformed the frailty index in predicting 30-day mortality. ^27^ Our findings are consistent with observations by Saetang et al., who reported superior performance of EuroSCORE II compared to the Modified Frailty Index-11 in predicting in-hospital mortality among similar patients. ^28^

Integrating frailty into predictive risk scores for mortality after cardiac surgery is increasingly recognized due to limitations of traditional risk models. Adding frailty has improved the predictive ability of EuroSCORE II and STS scores for 30-day mortality. ^29^^,^^30^ However, our study demonstrated that incorporating frailty indices did not enhance the predictive accuracy of evaluated models. This unexpected finding may be due to the specific frailty index chosen, the relatively low prevalence of frailty in our cohort, or the complex interplay between frailty and other risk factors. Further research is required to clarify the relationship between frailty, other risk factors, and mortality in elderly cardiac surgery patients.

Our study has several limitations. First, it was a single-center study with a relatively small sample size. Given the observed AUROC differences, our calculated statistical power was 62%, indicating a high risk of type II error. Additionally, the evaluated population was somewhat heterogeneous, including various surgical procedures such as double valve replacements and tricuspid valve replacements, which were not part of the original predictive models for the STS score and EuroSCORE II. Valve repairs were excluded because they are rarely performed at our center. The retrospective design may also introduce biases, as reliance on electronic records could result in coding errors, incomplete information, or selection bias in recorded data, potentially affecting the validity of findings. Our evaluation was limited to 30-day outcomes, since complete medium- and long-term follow-up data were unavailable. Although 30-day mortality strongly correlates with 12-month mortality, the performance of EuroSCORE II in predicting mortality declines over longer follow-up periods. ^31^^,^^32^

In conclusion, EuroSCORE II demonstrated the highest discriminatory ability for predicting 30-day mortality in older adults undergoing cardiac surgery. Incorporating frailty into a combined predictive model did not enhance performance compared to EuroSCORE II alone. These results underscore the need for future prospective, multicenter studies with larger sample sizes to further investigate the role of frailty in mortality prediction following cardiac surgery.
